# Community Based Project Work as a Teaching Tool: Students' Perception

**DOI:** 10.4103/0970-0218.45375

**Published:** 2009-01

**Authors:** Varsha M Vaidya, Jayashree S Gothankar

**Affiliations:** Department of Community Medicine, Bharati Vidyapeeth University Medical College, Pune, India

## Introduction

The medical college in general has the social accountability to produce a doctor who will take care of the health needs of the masses and not of the privileged few. It is up to the medical colleges to address this problem seriously and suggest measures.(1) Community-based training will enable students to understand the needs of communities and relate theoretical knowledge to practical training in a primary care context. This method of teaching and training the medical students has been recognized at various levels.(2)

The MCI curriculum, revised in 1997, has increased teaching learning hours in Community Medicine to emphasize community-based learning. Accordingly, a medical student has a 2½ month practical posting (forenoon-3 hours/day) in community medicine.(3) Per these guidelines, the department of Community Medicine of Bharati Vidyapeeth University Medical College (BVUMC), Pune gives community-based observational and/or interventional projects to fourth and sixth semester undergraduate medical students during their practical posting. These projects thus provided these students ample opportunities to interact with various sections of society.

This article discusses the feedback of these students on community-based projects with the objectives of assessing the students’ perception about community exposure and the level of satisfaction during community-based projects, assessing the type of study and term preferred by the students for doing projects, assessing their preference for the extension of the same project for more than one semester, and to study the strengths and weaknesses of the projects as perceived by the students.

## Materials and Methods

This study is based on feedback obtained from 169 students in the fourth and sixth semester at the end of their community-based projects. These community-based projects were completed within the time period of January 2007 to July 2007. The various project topics allotted were based on tuberculosis, HIV/AIDS, chikungunya, diabetes, malaria, fluorosis, old age homes, and breast cancer. The study area for these projects comprised of rural areas, urban slums in Pune, and secondary schools in Pune under the field practice area of BVUMC. A few of these projects were observational and a few were observational and interventional (health education sessions on various diseases in slums and schools, finding and eliminating breeding places of mosquitoes, etc.).

These 169 students were posted in the department in five practical posting batches of 4 weeks duration. Each batch was divided into five groups with five to six students in each group. One of the faculty members acted as a facilitator for each group.

Initially, these students were told to select a topic of public heath importance of their choice. The next 3 days were given for theoretical orientation to the subject with free access to the internet, including framing of aims and objectives of the project and developing tools for data collection.

Seven days were given for data collection in the community. During each of the remaining days, 30 minutes were utilized for project work like data compilation, data analysis, interpretation, and writing the project report. Presentation of project work was done on the last day for every batch and students also submitted a comprehensive report. Each teacher kept track of work for their respective students. Active involvement of students at every stage of the project was ensured. The students’ informed feedback was obtained at the end of posting and a certificate of participation was awarded to each student. Anonymity of the feedback given by the students was maintained and students were encouraged to express their views freely. The feedback form consisted of close-ended and open-ended questions. Close-ended questions were based on the level of satisfaction and the level of community exposure, which was graded from 0–5 (0-bad, 1-poor, 2-average, 3-satisfactory, 4-good, 5-excellent). Their preference for ‘ideal semester’ for doing project work and extension of the same project for more than one semester was obtained. Open-ended questions were based on the strengths and weaknesses of project work.

## Results and Discussion

A total of 78% of the students rated their level of satisfaction as good to excellent. A total of 70% of the students rated the level of community exposure as good to excellent followed by 24% who rated it as satisfactory. A total of 96% of the students mentioned their willingness to include a project as a routine part of their curriculum and a majority of the students considered the sixth semester as the ideal semester followed by the fourth semester for doing project work; 51% of the students mentioned their willingness to extend the same project to more than one semester.

The students' feedback on their preference for the type of project work was asked; 83% of students preferred a combination of observational and interventional study rather than doing only an observational study.

Open-ended questions for the strength of project work as perceived by the students revealed that 132 students received good exposure to the community in which they came across real life situations and they understood problems at a grass root level. They were also able to understand the lifestyle of people from low socio-economic environments. Of the 169 students, 81 students felt cooperation from the department was one of the strengths. A total of 54 students mentioned that working in groups or group interaction was a strength. Seventy students mentioned that the project helped them to develop good communication and problem solving skills. They found doing the project was interesting as there was lot of scope for creativity. Thirty students felt that doing community-based project work helped them to develop a sense of responsibility as future doctors.

A similar study done by Premarajan, *et al*. on undergraduate medical students in Eastern Nepal found that 85% of students felt the posting was useful in understanding the practical aspect of epidemiological studies and 67% of students rated the program from good to excellent.(4)

A total of 80% of students in a study done by JE Thistlethwaite on medical students commented that even 4 days of community based teaching had helped them realize the importance of understanding the patients’ side of the disease.(5)

A total of 68 students ranked finding the exact house of a patient living in the slum as the most important weakness. This experience helped them in understanding the social stigma associated with infectious diseases (tuberculosis, HIV/AIDS) in the form of fake names and fake addresses given by patients, the migration of patients, and incomplete data available at the urban health center. Inadequate time for the project was ranked as the most important weakness by 41 students, expenditure incurred in the project was ranked as the most important weakness by 35 students, and language barrier was ranked as the most important weakness by 24 students.

## Conclusion

Feedback from the students showed that they received good community exposure, which otherwise was not possible academically. It was a good learning experience for students. They understood the importance of correct and complete recording of data in the health information system, social stigma associated with certain diseases, communication skills in interview techniques, patient's knowledge, attitude, and practices towards certain infectious diseases, the lifestyle of people residing in the slums, and the care patients’ received in the family setup.

To sum up, the project work immensely helped the undergraduate students in understanding the subject of Community Medicine thoroughly. Thus, project work could be complementary to formal teaching in community medicine and appears to be a promising teaching tool.

## Recommendations

- The project work should be a routine part of the undergraduate curriculum of community medicine.
- The sixth semester seems to be an ideal semester for conducting projects.
- Standardization about semester, duration of project work, evaluation criteria, etc. needs to be completed for implementation in all medical colleges.
